# Quantile regression of tobacco tax pass-through in the UK 2013–2019. How have manufacturers passed through tax changes for different tobacco products?

**DOI:** 10.1136/tobaccocontrol-2020-055931

**Published:** 2020-10-22

**Authors:** Luke Brian Wilson, Robert Pryce, Rosemary Hiscock, Colin Angus, Alan Brennan, Duncan Gillespie

**Affiliations:** 1 School of Health and Related Research, University of Sheffield, Sheffield, UK; 2 Tobacco Control Research Group, University of Bath, Bath, UK

**Keywords:** taxation, tobacco industry, economics

## Abstract

**Background:**

The effectiveness of tax increases relies heavily on the tobacco industry passing on such increases to smokers (also referred to as ‘pass-through’). Previous research has found heterogeneous levels of tax pass-through across the market segments of tobacco products available to smokers. This study uses retail sales data to assess the extent to which recent tax changes have been passed on to smokers and whether this varies across the price distribution.

**Methods:**

We use panel data quantile regression analysis on Nielsen commercial data of tobacco price and sales in the UK from January 2013 to March 2019 combined with official UK tax rates and inflation to calculate the rate of tax pass-through for factory made (FM) cigarettes and roll your own (RYO) tobacco.

**Results:**

Following increases in the specific tax payable on tobacco, we find evidence of overshifting across the price distribution for both FM and RYO. The rate of the overshift in tax increased the more expensive the products were. This was consistent for FM and RYO. Additionally, our findings suggest that the introduction of standardised packaging was not followed by changes in how the tobacco industry responded to tax increases.

**Conclusions:**

Following the repeated introduction of increases in specific tobacco tax as well as standardised packaging, we show that the tobacco industry applies techniques to keep the cheapest tobacco cheaper relative to the more expensive products when passing on tax increases to smokers.

## Introduction

In the UK, the tax structure applied to tobacco products includes three types of tax (excluding heated tobacco products). First, a specific tax, this is a fixed amount per 1000 factory made (FM) cigarettes or per 1000 g of roll your own (RYO) tobacco. These lump-sum rates are different between FM and RYO. Second, a tobacco ad valorem tax, that is only applied to FM cigarettes, which is a proportion of the retail price (16.5% of the recommended retail price, RRP). This tax rate was set in March 2011 and has not changed in the time frame of this study. The ad valorem tax is only applied to FM cigarettes in the UK. Finally, there is a general value-added tax (VAT), which is a UK sales tax that is applied to most goods and services. Throughout the period of analysis, the rate of VAT has remained unchanged at 20% value-added on top of the retail price. Table 1 shows two examples of RRPs for FM and RYO broken down into the various tax elements payable on each product as of October 2018.

**Table 1:** Breakdown of taxes applied to factory made cigarettes and roll your own tobacco

	Factory made (FM) cigarettes 20 sticks (£)		Roll your own (RYO) tobacco 30 g (£)	
**Price (RRP**)	**8.50**	**12.00**	**14.50**	**15.19**
VAT at standard rate (20%)	1.42	2.00	2.42	2.53
Ad valorem tax (at 16.5%)	1.40	1.98		
*Specific tax for 1000 cigarettes/g*	*228.29*	*228.29*	*234.65*	*234.65*
Specific tax for 20 cigarettes/30 g	4.57	4.57	7.04	7.04
**Total tax**	**7.39**	**8.55**	**9.46**	**9.57**
Tax (% of RRP)	87	71	65	63
**Net revenue**	**1.11**	**3.45**	**5.04**	**5.62**

The specific tax for 1000 cigarettes/g, ad valorem, and VAT rates as of October 2018.

RRP, recommended retail price; VAT, value-added tax.

In May 2017, the UK introduced a minimum excise tax (MET) on FM cigarettes. The MET, in effect, sets a price floor for the added total of two of the components (specific and tobacco ad valorem, but not VAT) charged to each product. If the total amount of specific and ad valorem tax payable on each product falls below the MET, then the tax payable is that of the MET rather than the sum of the specific and ad valorem tax elements.

A substantial body of evidence has documented the effectiveness of tobacco tax increases on reducing tobacco prevalence,^1–5^ participation^6–9^ and initiation^10–12^ as well as raising government revenue. However, the effectiveness of tax increases on health-related outcomes relies heavily on the tobacco industry (TI) passing on such tax increases to smokers, also known as tax pass-through.^13–15^ Previous work revealed that, between 2000 and 2015 in the UK, the TI passed on tax increases to smokers differently depending on the market segment of the product. They did this by absorbing tax increases on the cheapest products in the price distribution (undershifting), while simultaneously increasing the price, above the tax increase, of the relatively more expensive products (overshifting) in order to try and maximise revenue or maintain profits.^13 16^ Research conducted for the period before standardised packaging in the UK found that the TI uses a variety of strategies to keep the cheapest FM cigarettes and RYO tobacco cheap. This includes the use of price-marked packaging, smaller pack sizes as well as dropping net revenue per pack by 18 pence per pack at the time of a UK budget, absorbing the tax increase and accentuating the price gap between premium and ultra-low price products.^13 16^

When examining the claims made by the TI that tax increases will only increase the demand for illicit tobacco, further research in the UK found that the TI increased its prices beyond that required by tax changes, even when tax changes were larger or unexpected, thus increasing revenue for all products except for FM sub value 19 stick.^14^ On all but the cheapest brands, the authors found that these proportions were approximately 55% (industry price increase) and 45% (tax).^17^

The UK government additionally introduced standardised packaging for all FM and RYO tobacco.^18^ This required the removal of colourful expression of brands as well as the standardisation of other key features of the packaging such as the dark brown colour packaging (Pantone 448C), font, health warnings and imagery and pack size (minimum 20-stick for FM and 30 g for RYO). This came partially into force on 20 May 2016, in which all newly manufactured or imported FM or RYO products had to comply with the standardised packaging. To allow for the sale of existing stock, full implementation, in which all products had to comply with standardised packaging, came into force on 20 May 2017. Therefore, the appearance of standardised packs in our period of analysis occurs gradually.

Contrary to the initial arguments made by the TI, researchers found that there was no long-term lowering of tobacco prices following the introduction of standardised packaging and MET.^15 19^ They found that tax changes following the implementation of these policies were more widely and quickly passed on to smokers in the form of higher prices for FM and RYO. However, sales volumes of RYO continued to increase throughout the study period, perhaps because RYO remains a less expensive means of smoking tobacco.

This study aims to use market research sales data at stock keeping unit (SKU) level to investigate the extent to which tobacco tax changes are passed through to smokers in the UK. Our period of study is January 2013 to March 2019, which covers a longer period both prestandardised and poststandardised packaging than previous work.^15^ We adjust for inflation over the period, and compute expected counterfactual prices if all tax changes had been passed through to smokers exactly. The statistical analysis extends previous work by applying panel data quantile regression methods to the monthly pricing data in order to produce estimates of undershifting or overshifting of the tax changes at different price points covering the cheapest to most expensive products. We examine the extent of tax pass-through over the full period and compare differences prestandardised and poststandardised packaging introduction. As of 2020, 14 countries have implemented standardised packaging and four more have passed legislation, so our results are globally relevant.

## Methods

### Nielsen Scantrak data

We used monthly UK data from Nielsen Scantrak from January 2013 to March 2019. Nielsen is a global measurement and data analytics company that provides data on the tobacco markets worldwide. Scantrak data are collected at the point of sale in which an electronic barcode of FM cigarettes or RYO tobacco is scanned via electronic point of sale system during a purchase at a participating retailer. Nielsen collect sales data from 87% of Great Britain’s supermarkets, 15% of its convenience stores (including 83% of supermarket-owned convenience stores, 59% of petrol station forecourts, 6% of convenience store chains and 4% of independent retailers) and 17% of Northern Irish stores with grocery sales (Northern Ireland represents 2.8% of the UK population). The sampling includes 100% of the big four UK supermarkets (Tesco, Sainsbury, Asda and Morrisons). For other stores, stratified random sampling with replacement is used. Nielsen then models this Scantrak data to provide a data set that is representative of the UK as a whole using expansion factors based on the region, shop type and shop company.^20^

Nielsen provided the research team with a data set in which each row is a specific SKU in a specific month. Nielsen calculates the price paid per pack for each SKU as well as the volume of sales (number of sticks sold for each SKU). Following on from the previous literature,^13 21–23^ we focus on a four-level product hierarchy within the TI: brand (eg, John Player), brand family (eg, John Player Special), brand variant (eg, John Player Special Real Blue), and the lowest available indicator, SKU (eg, John Player Special Real Blue King Size x20).

As well as providing identifiers used in the four-level product hierarchy, for each SKU, Nielsen provides the number of sticks sold per pack for FM and the grams per pack for RYO, whether the SKU is ‘price-marked’ or whether the SKU has standardised packaging. For sample design reasons, Nielsen recommended only analysing SKUs that have a market share greater than 0.8%. This also follows previous literature that has used similar data.^13–15^

FM products were classified as being in either ‘fully branded packaging’ which is the old-style colourful packaging sold in a variety of stick sizes, or standardised packaging^19^ which was introduced in the UK between May 2016 and May 2017. Branded packaging could further be either price-marked (price printed on the packaging) or non-price-marked. The Nielsen data do not specify whether RYO was in branded or standardised packaging, therefore we classified RYO tobacco as standardised packaging if it was 30 g and appeared in the data post the introduction of standardised packaging.

### The counterfactual expected price

For every SKU in our analytic sample, we constructed a monthly time series of the counterfactual expected price based on the ‘baseline’ actual price at the time point when the product is first observed in the Nielsen data, and the expected impact of both inflation and tax changes in subsequent months, assuming all tax increases had been fully passed through to the smoker. table 2 summarises all the specific tax changes that have occurred between 2013 and 2019. We then compare this to the actual observed prices at which that product was sold in subsequent months.

**Table 2:** Tobacco-related tax changes during the period of analysis

Tax	March 2012	March 2013	March 2014	March 2015	March 2016	March 2017	May 2017	November 2017	October 2018
Panel A: Specific excise tax for each tobacco type over time									
Factory made*	167.41	176.22	184.10	189.49	196.42	207.99	207.99	217.23	228.29
Minimum excise tax†							268.63	280.15	293.95
Roll your own‡	164.11	172.74	180.46	185.74	198.10	209.77	209.77	221.18	234.65
Panel B: Change in specific excise tax from previous period									
Factory made		8.81	7.88	5.39	6.39	11.57	0.00	9.24	11.06
Roll your own		8.83	7.72	5.28	12.36	11.67	0.00	11.41	13.47
Panel C: Percentage change in specific excise tax compared with previous period									
Factory made		+5.26	+4.47	+2.93	+3.66	+5.89	0.00	+4.44	+5.09
Roll your own		+5.26	+4.47	+2.93	+6.65	+5.89	0.00	+5.44	+6.09
Panel D: Ad valorem and VAT tax rates over time									
Ad valorem tax§ (%)	16.5	16.5	16.5	16.5	16.5	16.5	16.5	16.5	16.5
VAT (%)	20	20	20	20	20	20	20	20	20

*Specific tax £ per 1000 cigarettes.

†Minimum excise tax introduced for factory made cigarettes: specific tax plus ad valorem tax (16.5%) of RRP.

‡Specific tax £ per 1000 g.

§Applied to FM cigarettes only.

FM, factory made; RRP, recommended retail price; VAT, value-added tax.

One of the key variables of interest in our study is the expected price per stick E[Price] . This is the price per stick in pence for each SKU *i* assuming that there is full tax pass-through at the time of the specific tax change *t*. In order to calculate the expected price per stick, we first calculated the net revenue from each pack; this was done by removing all tobacco taxes applied to that product at a point in time.

The tax applied to each SKU varies depending on whether they are FM cigarettes or RYO tobacco. The tax duty on FM cigarettes consists of three elements: a specific component which is an ad quantum tax per 1000 FM cigarettes, an ad valorem component which is a percentage of the retail price and the UK value-added sales tax VAT. RYO tobacco has only two components: a specific tax per 1000 g of RYO tobacco and VAT. During our period of analysis, the ad valorem component for FM cigarettes and VAT remained unchanged, details of the changes in the specific tax and MET are summarised in table 2.

Once we calculated the ad valorem tax for FM cigarettes, we converted all taxes payable for each SKU into per stick and calculated the evolution of expected price per stick for all tobacco products. Following existing literature, as well as best current estimates, we assumed that RYO tobacco is 0.5 g per stick.^13 14 16^ The initial expected price per stick, $E[Price_{it}]$ takes the value of the observed price in the first time period it appears in the data. To construct the evolution of $E\left[Price_{it}\right]$ over time, we removed the amount of the specific tax $\left(Duty_{it=1}\right)$ , VAT and $advaloremtax_{it=1}$ for FM cigarettes that would have been due at time *t*=1, this leaves only net revenue, see Equation 1.

$$\begin{array}{c} NetRevenue_{it=1}=\frac{RetailPriceperStick_{it=1}-SpecificDutyperStick_{it=1}}{VAT{\%}_{t=1}}\\ -AdValoremTaxperStick_{it=1}\left(B1\right) \end{array}$$

Net revenue is the money the TI retains from its sales once all taxes and VAT have been paid. This forms a ‘baseline’ price, which is then inflated to real terms using the Retail Price Index $\left({RPI}_{t}\right)$ as this is used to set the path for most specific tax rates in the UK.^24^ RPI is a measure of inflation published monthly by the Office for National Statistics. It measures the change in the cost of a representative sample of retail goods and services. The UK Treasury uses RPI for various index-linked tax rises. This inflated baseline price is then updated over the course of the time frame in the data to reflect the incremental change in specific tax in each following time period. Similarly, if a product’s price increases exactly in line with specific tax, VAT and inflation, then its expected price is equal to observed price. Our estimated equation to get expected price becomes as follows:

$$\begin{array}{c} E[Price_{it}]=((NetRevenue_{it})\times RPI_{t}+SpecificDutyStick_{it})\\ \times (VAT{\%}_{t})+AdValorenTaxperStick_{it}(B2) \end{array}$$

### Panel data quantile regression strategy to estimate tax pass-through across the price range

Similar to evidence examining tax pass-through in the alcohol industry,^25^ we exploit the panel nature of the price data and adopt a quantile regression approach.^26^ Rather than focusing only on the predicted mean of the dependent variable, as in classical linear regression, quantile regression focuses on quantiles which refer to defined points in the price distribution. For example, the 0.50 quantile is the median and 0.05 is the fifth percentile (the cheapest) of the distribution. This allows for the flexibility for modelling the entire distribution of observed prices as the dependent variable. This methodology provides a framework for investigating differential tax pass-through for price points across the entire price distribution.

The basic version of our model is as follows:

$$\begin{array}{c} ObservedPrice_{it}=\beta_{0}+\beta_{1,\theta }ExpectedPrice_{it}+\varepsilon_{it,\theta } \end{array}$$

Where $ObservedPrice_{it}$ is the observed price per stick of SKU *i* at time t in the Nielsen data and $ExpectedPrice_{it}$ is the price per stick calculated counterfactual price assuming a full tax pass-through. These are both weighted using the volume of sticks sold for each SKU supplied by Nielsen.

Following previous literature in the alcohol market,^25^ we consider 11 quantiles (0.05, 0.15, 0.25, 0.35, 0.45, 0.5, 0.55, 0.65, 0.75, 0.85, 0.95) which includes the median θ=0.50 to try and fully capture the price distribution of tobacco. We run the model for FM and RYO separately.

If tax changes are fully passed onto smokers across the price distribution then, for all quantiles, the estimated $\beta_{1}$ coefficient of a given SKU should equal 1. If $\beta_{1}$ is less than 1, this is an example of undershifting, and the producer is losing some revenue and bears some of the burden of the tax change. If $\beta_{1}$ is great than 1, this represents overshifting and the smoker is paying more than the 100% tax pass-through expected price given the tax change, and the TI or retailer is gaining additional revenue.

## Results

### Evolution of prices over time

**Figure 1:**
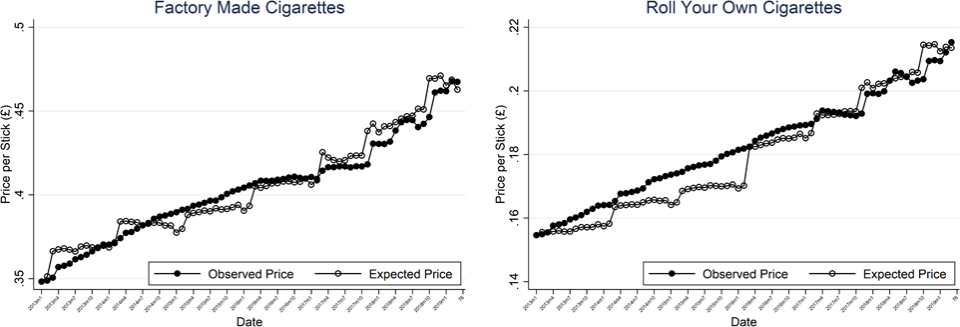
Evolution of prices over time.

Figure 1 illustrates the changes in observed price per stick and the calculated counter factual expected price per stick for FM and RYO considering the expected impact of both inflation and specific tax changes in subsequent months. We weight these using the market share of the SKU (% of sticks sold).

In panel A, broadly, FM observed average prices track with expected counterfactual prices, though at times the observed was above and at other times below. This was more prominent when specific tax changes occurred, the observed prices took a few months to catch up. With respect to RYO in panel B, observed prices are above the expected counterfactual prices slightly more often than for FM suggesting that tax pass-through might be slightly higher for RYO than FM.

### Descriptive statistics on quantiles of price

Table 3 summarises the observed price paid per stick for the upper bound of each quantile band (θ) as well as the percentage sold in each of the 11 quantile bands for FM cigarettes and RYO tobacco. Each θ quantile can be interpreted as the % of SKUs in that price band. For example, θ≤0.05 equates to 5% of SKUs in our data set are sold below 33 p per stick. Table 3 illustrates the difference in price between the cheapest products available (θ≤0.05) and the most expensive products (θ>0.95). This also signifies the large differences in price between FM cigarettes and RYO tobacco, therefore, the quantile bands for FM and RYO are on different scales.

**Table 3:** Quantiles of price paid per stick and volume of sales

	Factory made cigarettes		Roll your own tobacco	
	Price per stick (£)*	Market share of sticks sold (%)†	Price per stick (£)*	Market share of sticks sold (%)†
0.95> θ	0.486	9.42	0.215	22.37
0.85 < θ≤0.95	0.466	18.23	0.205	13.05
0.75 < θ≤0.85	0.424	10.70	0.194	9.94
0.65 < θ≤0.75	0.412	16.11	0.186	13.79
0.55 < θ≤0.65	0.407	11.44	0.180	11.58
0.50 < θ≤0.55	0.392	1.65	0.174	7.99
0.45 < θ≤0.50	0.385	2.35	0.171	7.08
0.35 < θ≤0.45	0.377	7.44	0.168	3.75
0.25 < θ≤0.35	0.365	6.77	0.163	3.83
0.15 < θ≤0.25	0.352	6.76	0.159	3.37
0.05 < θ≤0.15	0.341	6.88	0.153	2.96
θ≤0.05	0.329	2.25	0.142	0.31

*Price per stick refers to the upper bound of each quantile band (θ).

†Volume sold is the percentage sold in this category at this price band as a percentage of all FM or RYO sold in this category. Price distribution for a specific product class captures the prices of all products falling within the category. Volume sold refers to the % sold in each price band, due to rounding, total sales may not sum to 100%.

FM, factory made; RYO, roll your own.

The cheapest RYO (assuming 0.5 g of tobacco per stick) is 14.2 p per stick, while the cheapest FM stick is 32.9 p. There is also a large difference in price in the most expensive products available. The upper bound for FM is 58.6 p, while the most expensive RYO stick is 23.5 p, which is cheaper than the cheapest FM cigarettes available. Here we also present the volume of sales for each quantile. For both products, the volume of sales increases the further up the price distribution.

### Estimates of pass-through using panel data quantile analysis

**Figure 2:**
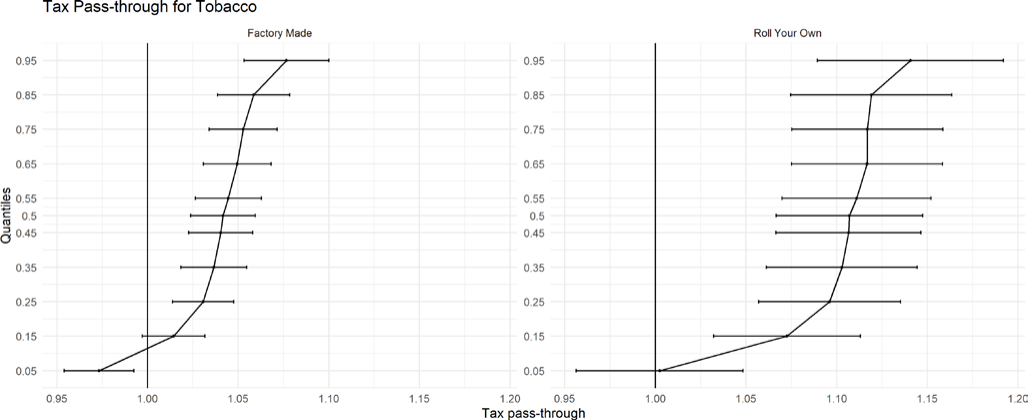
Tax pass-through for tobacco.

Figure 2 illustrates the tobacco-specific tax pass-through estimates for all FM cigarettes and RYO tobacco (see appendix for regression parameters). At the lowest end of the price distribution (θ≤0.05), our results show that there is undershifting for the cheapest FM cigarettes 0.97 (0.95, 0.99). On the other hand, at (θ≤0.05), the results suggest very close to 100% tax pass-through for RYO.

On average, the median SKU (θ=0.50) of FM cigarettes pass-through specific tax changes slightly above the full tax pass-through at 1.04 (1.02, 1.06). However, for RYO this is somewhat higher at 1.10 (1.07, 1.15), suggesting that the TI passes through the change in RYO tax at a higher rate than FM.

For the remaining quantiles, there is evidence to suggest clear overshifting of tax on to smokers, that is, prices are going up more than the specified tax increase. The interesting narrative however is that the overshift is smallest at the lower end of the price distribution. At θ=0.25, the rate of tax pass-through is 1.03 (1.01, 1.04) for FM and 1.10 (1.06, 1.13) for RYO, while at the higher end of the price distribution (θ=0.75) this increases to 1.05 (1.03, 1.07) and 1.12 (1.08, 1.16) for FM and RYO, respectively.

These estimates show that while the TI is increasing the prices of their products beyond that of the tax increase, they are still trying to keep the cheapest products cheap relative to the more ‘premium’ and more expensive products. This outcome is consistent for both FM and RYO tobacco. As a result, this widens the gap between the cheapest and more expensive products as prices have increased at a greater rate at the higher end of the price distribution.

### Standardised packaging

**Figure 3:**
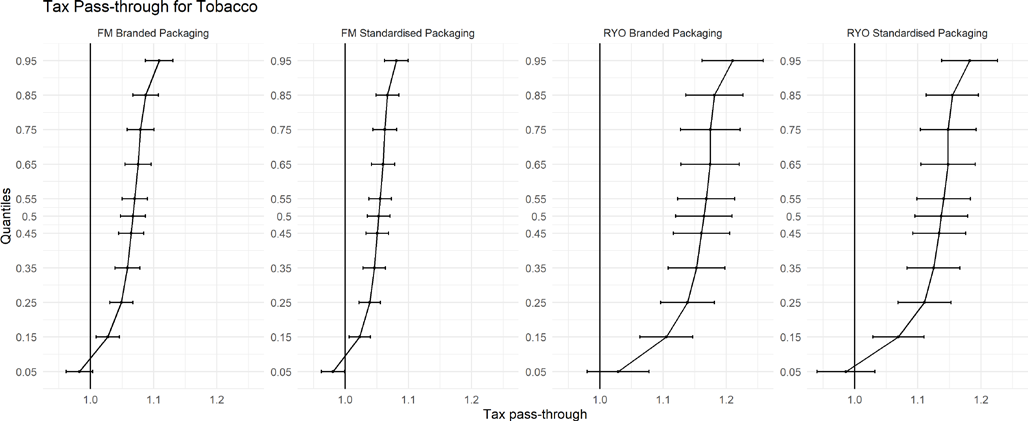
Tax pass-through for tobacco prestandardised and poststandardised packaging. FM, factory made; RYO, roll your own.

Our period of analysis covers both prestandardised and poststandardised packaging and the introduction of the MET. In figure 3, we split our sample into ‘branded packaging’ which is the old-style, colourful packaging which was sold in a variety of stick sizes and the new style ‘standardised packaging’. We estimate separately the tax pass-through of the specific tax increases prestandardised and poststandardised packaging. We find that the rates of tax pass-through for FM and RYO do not vary across the introduction of standardised packaging. There is marginally lower tax pass-through after standardised packaging compared with before, for example, 1.04 (1.02, 1.06) compared with 1.02 (1.01, 1.05) at θ=0.50 for FM product, however, they are not statistically different to each other. This pattern is consistent in the two periods.

## Discussion

This paper uses quantile regression for panel data to estimate the rate of tax pass-through in the market for tobacco. We find that the TI predominantly overshifts tax in the form of higher than expected prices. Furthermore, we find that the magnitude of the overshift is greater at the more expensive end of the price distribution, relative to the cheaper products available. This is consistent for both FM and RYO.

Research conducted prestandardised packaging in the UK found that the TI uses a variety of strategies to keep the cheapest FM cigarettes and RYO tobacco cheap.^13 14 16 18^ We find that our results are consistent with the previous literature as the rate of the overshift is greater the higher up the price distribution.

One of the main arguments the TI made in its opposition to the introduction of standardised packaging was that it would result in the commoditisation of tobacco and reduce prices. Previous evidence showed the introduction of standardised packaging did not lead to a long-term decline in cigarette prices.^15^ We also show that the rates of tax pass-through for branded versus standardised packaging do not differ significantly since the implementation of standardised packaging and have a longer follow-up period.

Another strength of our analysis is our use of quantile regression for panel data to estimate the rate of tax pass-through in the market for tobacco. Unlike previous analyses which used a price segmentation approach via a commercial literature review, we were able to break the market into consistent, exogeneously defined, quantiles. That both approaches revealed similar findings provides triangulation.

While we provide new evidence regarding the extent of tax pass-through by the TI, our work is not without limitations. Due to commercial sensitivity, we are unable to obtain the exact ad valorem rate payable or RRP suggested by the TI for each SKU in the Nielsen data. Instead, we use similar methods from the previous literature^13–15^ as well as conversations with Her Majesty’s Revenue and Customs to calculate the ad valorem tax for each SKU. Following this guidance, we are confident that our estimates are close to those paid.

There is some further potential thinking to be done about how these findings are related to understanding of price elasticities for tobacco. Most of the current estimates on price elasticity estimates for tobacco examine average mean changes in sales or purchasing and there is little published evidence on differential price elasticities at different price points across the price distribution or across product segments. Our research illustrates that the TI passes on tax increases heterogeneously across the price distribution; an extension to this can be to examine how smokers respond to the price increases they face.

## Conclusions

Our results show that tax increases introduced in the UK lead to increases in the price of tobacco paid by smokers across the price distribution and therefore support the extensive evidence on the effectiveness of duty increases on reducing tobacco consumption and prevalence. However, our results also indicate that the TI keeps the cheaper tobacco products cheaper than the more ‘premium’ products by overshifting taxes at a lower rate.

**What this paper adds**

- Previous work in the UK has shown that the tobacco industry (TI) passed tax duty increases on to smokers at different magnitudes depending on the market segment of the product.
- The TI did this by absorbing tax increases on the defined ‘subvalue’ and ‘value’ products, while simultaneously increasing the price, above the tax increase, of the relatively more ‘premium’ products in order to maximise revenue.
- What is not as clear is how the rate of tax pass-through, following specific tax increases, varies across the price distribution of factory made cigarettes and roll your own tobacco as tobacco product segmentation overlaps across the price distribution.
- We create a counterfactual expected price taking into consideration specific tax increases and inflation in the UK and undertake quantile regression of this against market research pricing data to estimate tax pass-through of factory made cigarettes and roll your own tobacco across the price distribution of prices faced by smokers.
- The statistical analysis in this paper extends previous work by applying panel data quantile regression methods to the monthly pricing data in order to produce estimates of undershifting or overshifting of tobacco tax changes at various price points across the entire distribution, covering the cheapest to most expensive products available to smokers.
- Our evidence shows that the TI overshifts tax increases at a higher rate for the more expensive products relative to the cheapest products at the lower end of the price distribution. This is consistent for both factory made and roll your own tobacco.

10.1136/tobaccocontrol-2020-055931.supp1 Supplementary data

## Data Availability

Data may be obtained from a third party and are not publicly available. We do not have the right to share the Nielsen data. Researchers must contact Nielsen directly to purchase a copy of the data. We are able to make the instructions and code available by posting detailed documentation on our code and programs, instructions on how researchers can obtain the data, and online readme files.
