# Supplementary material for: Quantile regression of tobacco tax pass-through in the UK 2013–2019. How have manufacturers passed through tax changes for different tobacco products?
Source: Tob Control. 2020 Oct 22;30(e1):e27–32. doi: 10.1136/tobaccocontrol-2020-055931 (PMC8606450; doi:10.1136/tobaccocontrol-2020-055931)

Quantile regression of tobacco tax pass-through in the UK 2013 - 2019.

How have retailers passed tax changes for different tobacco products?

SUPPLEMENTARY MATERIALS

Luke B. Wilson[1]

Robert Pryce[1]

Rosemary Hiscock[2]

Colin Angus[1]

Alan Brennan[1]

Duncan Gillespie[1]

[1] Sheffield Alcohol Research Group, School of Health and Related Research, University of Sheffield, Sheffield, S1 4DA, UK.

[2.] Tobacco Control Research Group, University of Bath, Claverton Down, Bath, BA2 7AY, UK.

Corresponding author email: l.b.wilson@sheffield.ac.uk

Corresponding author telephone: 0114 222 0815

Corresponding author Orchid ID: 0000-0001-5769-5729

Keywords: Tobacco, Tax pass-through, Tax policy

JEL Codes: I13, I18, H22

Appendix

Box 1: Box 1 which is currently in the submitted draft can be removed and added to the appendix.

Table S1: Point estimates of panel data quantile regression from Figure 2

Quantile	Factory Made	Roll Your Own
0.05	0.973	1.002
	(0.01)	(0.023)
0.15	1.014	1.073
	(0.009)	(0.02)
0.25	1.031	1.096
	(0.008)	(0.019)
0.35	1.037	1.103
	(0.009)	(0.02)
0.45	1.04	1.107
	(0.009)	(0.02)
0.50	1.042	1.107
	(0.009)	(0.02)
0.55	1.045	1.111
	(0.009)	(0.02)
0.65	1.05	1.117
	(0.009)	(0.02)
0.75	1.053	1.117
	(0.009)	(0.021)
0.85	1.059	1.119
	(0.01)	(0.022)
0.95	1.077	1.141
	(0.012)	(0.026)

Figure S1: Main Result estimated using CPIH

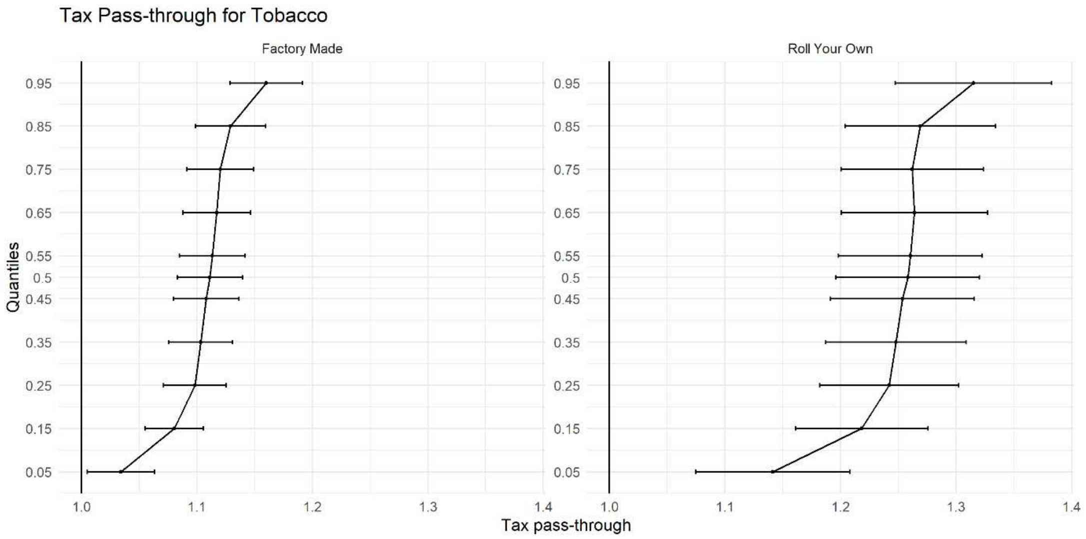

Figure S2: Method for calculating the ad valorem element for Factory Made Cigarettes

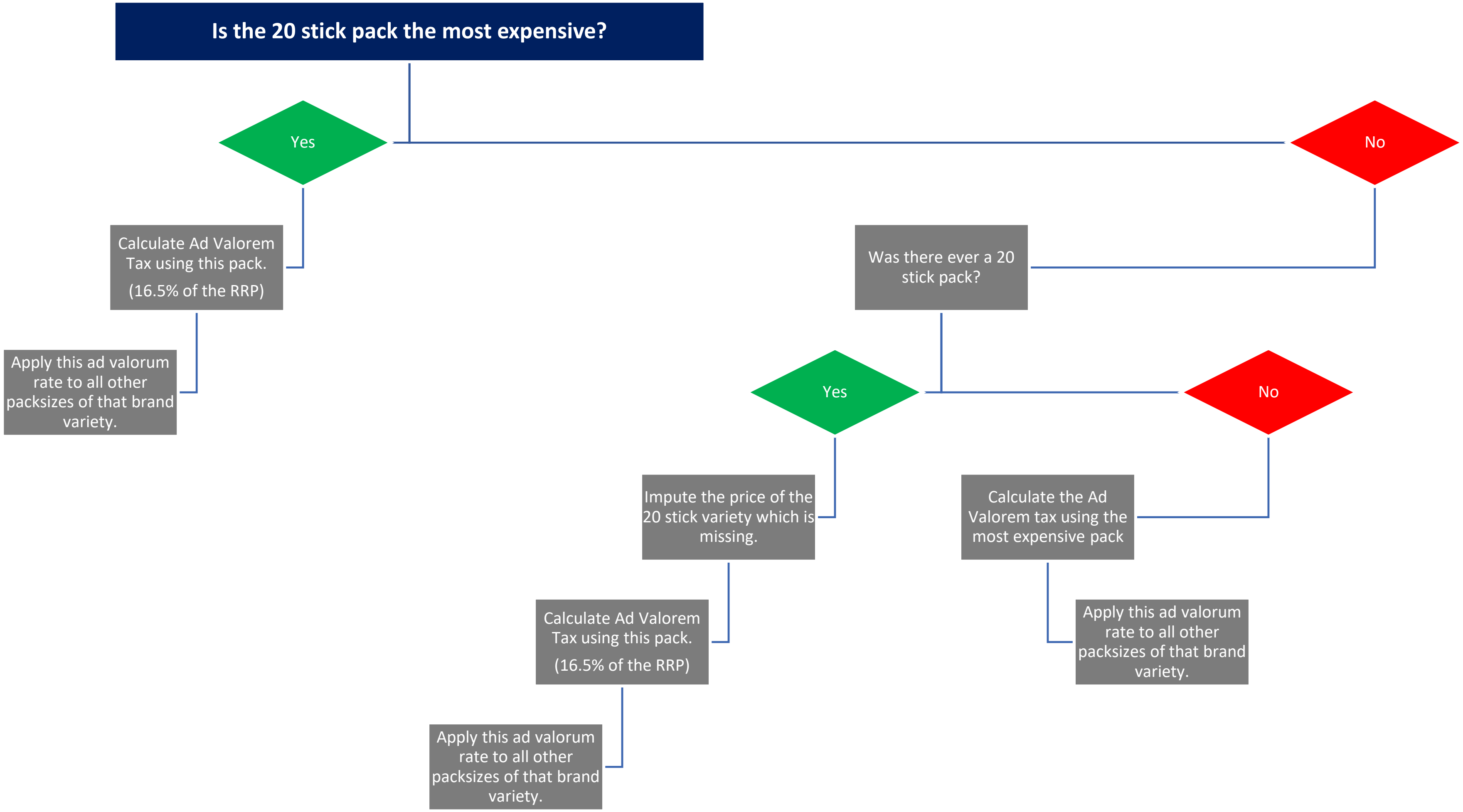

Supplement: Supplementary data [file tobaccocontrol-2020-055931supp001.pdf]
